# The Role of S6K1 in Aging and Alzheimer’s Disease: Mechanistic Insights and Therapeutic Potential

**DOI:** 10.3390/ijms26135923

**Published:** 2025-06-20

**Authors:** Salvatore Oddo, Marika Lanza, Giovanna Casili, Antonella Caccamo

**Affiliations:** Department of Chemical, Biological, Pharmaceutical and Environmental Sciences, University of Messina, Viale F. Stagno d’Alcontres 31, 98166 Messina, Italy; salvatore.oddo@unime.it (S.O.); marika.lanza@unime.it (M.L.); giovanna.casili@unime.it (G.C.)

**Keywords:** aging, Alzheimer’s disease, S6K1

## Abstract

Aging is the greatest risk factor for Alzheimer’s disease (AD), but the mechanisms connecting the two remain unclear. The mammalian target of rapamycin (mTOR) pathway, particularly its downstream effector S6 kinase 1 (S6K1), has emerged as a key regulator of aging and neurodegeneration. S6K1 controls translation, autophagy, and mitochondrial function—processes disrupted in both aging and AD. This review examines how S6K1 influences mitochondrial metabolism, autophagy, and metabolic dysfunction in aging. We also discuss its role in the nervous system, including effects on synaptic plasticity, memory, glial activation, and neuroinflammation. In AD, S6K1 contributes to amyloid and tau pathology by regulating translation of BACE1 and tau, and its hyperactivation is linked to synaptic deficits and cognitive decline. We further explore therapeutic strategies targeting S6K1, which have shown benefits for lifespan extension and neuroprotection in preclinical models. Finally, we consider the emerging link between S6K1 and necroptosis, a form of programmed cell death implicated in AD-related neuronal loss. Together, these findings highlight S6K1 as a promising target for interventions aimed at slowing aging and mitigating AD pathogenesis.

## 1. Introduction

Aging is the greatest risk factor for Alzheimer’s disease (AD), a neurodegenerative disorder characterized by progressive cognitive decline, synaptic dysfunction, and neuronal loss) [1]. Despite decades of research, the molecular mechanisms linking aging to AD pathogenesis remain unclear. One central pathway implicated in both aging and AD is the mammalian target of rapamycin (mTOR) signaling, which governs cellular metabolism, protein synthesis, and autophagy [2,3,4]. The ribosomal protein S6 kinase 1 (S6K1), a serine/threonine kinase that regulates translation and metabolic homeostasis, is a key downstream effector of mTOR [5]. Increasing evidence suggests that S6K1 plays a pivotal role in aging and neurodegeneration, making it a compelling target for therapeutic intervention [6,7,8]. S6K1 plays a primary role in protein synthesis and cellular growth, acting through phosphorylation of ribosomal protein S6 and other targets involved in translation [9,10,11,12]. However, beyond its classical function in translation control, S6K1 has emerged as a critical regulator of lifespan and health span. To this end, complementary studies in animal models, including mice and Drosophila, have demonstrated that the inhibition of S6K1 extends lifespan and improves metabolic function [7,13]. The effects of S6K1 on aging are thought to be mediated through its interactions with mitochondrial homeostasis, oxidative stress responses, and autophagy, processes that are also dysregulated in AD [14,15,16]. Given the overlapping molecular mechanisms that govern aging and AD, S6K1 represents an intriguing link between these processes. Alterations in mTOR/S6K1 signaling have been observed in AD, influencing key pathological features such as amyloid-β (Aβ) accumulation, tau hyperphosphorylation, and synaptic dysfunction [17,18,19,20,21,22]. Understanding the role of S6K1 in AD pathogenesis may provide novel insights into the molecular basis of neurodegeneration and identify new therapeutic avenues. This review will explore the function of S6K1 in aging and its implications in AD, with a particular focus on the mechanistic pathways through which S6K1 influences neurodegeneration and the potential for targeting S6K1 in AD therapy.

## 2. S6K1 and the Biology of Aging

### 2.1. Regulation of S6K1 Activity in Aging

**Figure 1:**
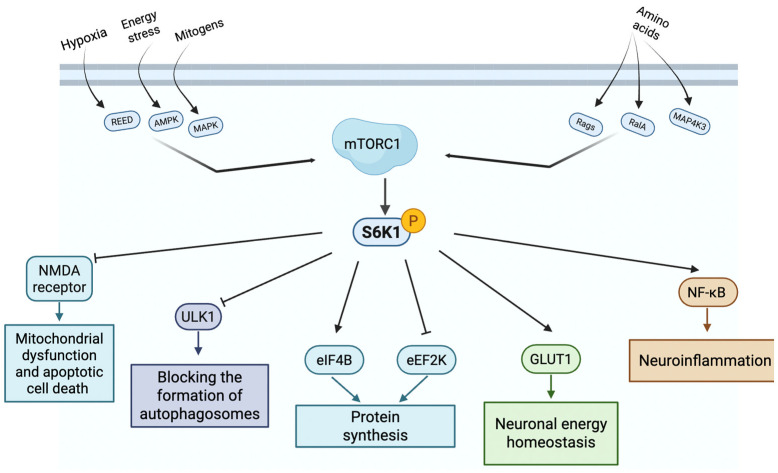
Schematic representation of S6K1 pathway.

S6K1 is activated by mTOR complex 1 (mTORC1) in response to growth factors, nutrients, and cellular energy status (Figure 1) [23]. mTORC1 phosphorylates S6K1 on its hydrophobic motif (T389), which primes it for full activation by additional phosphorylation from 3-Phosphoinositide-dependent protein kinase 1 (PDK1) at the activation loop [24,25]. Active S6K1 phosphorylates ribosomal protein S6, eukaryotic initiation factor 4B (eIF4B), and eukaryotic elongation factor 2 kinase (eEF2K; Figure 1), all of which promote protein translation [26]. S6K1 not only regulates cellular metabolism and protein synthesis but also plays a critical role in cell cycle progression, particularly at the G1/S transition [27,28]. Through its downstream targets, S6K1 promotes ribosomal biogenesis and the translation of key cell cycle regulators, such as cyclin D1 and cellular myelocytomatosis oncogene (c-Myc), both of which are essential for G1-phase progression and S-phase entry [27,28]. While this regulation is essential for cellular growth and development, sustained S6K1 activation has been linked to aging-associated pathologies, including insulin resistance, mitochondrial dysfunction, and inflammation [29,30].

### 2.2. S6K1 and Mitochondrial Function in Aging

Mitochondrial function is pivotal in cellular energy production and metabolic regulation, both of which are crucial during the aging process. Strong evidence indicates that S6K1 significantly influences mitochondrial dynamics and function, thereby impacting cellular aging [15,31,32,33]. S6K1 plays a critical role in maintaining mitochondrial morphology by modulating the balance between mitochondrial fusion and fission. For example, studies in mouse embryonic fibroblasts (MEFs) lacking S6K1 have demonstrated increased mitochondrial fragmentation. This phenotype is associated with elevated levels of dynamin-related protein 1 (Drp1) and its active phosphorylated form at Ser616, both in whole-cell extracts and mitochondrial fractions [15]. The alterations in mitochondrial morphology due to S6K1 deficiency have significant metabolic consequences. In S6K1-knockout MEFs, there is an observed increase in glycolysis and mitochondrial respiratory activity. However, oxidative phosphorylation (OxPhos) ATP production remains unaltered [15]. Notably, inhibition of Drp1 with Mdivi1, a Drp1 inhibitor, enhances OxPhos ATP production and reduces mitochondrial membrane potential, suggesting that the fragmented mitochondria in S6K1-deficient cells are less efficient in ATP production. This implies that S6K1 modulates energy metabolism by influencing mitochondrial respiratory capacity and morphology [15]. Similar findings have been reported in human cell lines. Stable knockdown of S6K1 in HeLa cells leads to noticeable changes in mitochondrial morphology, characterized by increased fragmentation. This is accompanied by upregulation of mitochondrial fission proteins, including Drp1 and mitochondrial fission 1 protein (Fis1) [33]. Furthermore, S6K1-depleted HeLa cells exhibit elevated oxygen consumption rates and enhanced ATP production in both cytoplasmic and mitochondrial compartments. Despite this increase, the relative ratio of mitochondrial ATP to cytoplasmic ATP is decreased, indicating a shift in energy production dynamics [33]. Additionally, induction of mitophagy is observed, suggesting that S6K1 influences mitochondrial quality control mechanisms. Further strengthening the link between mitochondrial function and S6K1, this kinase suppresses the expression and activity of peroxisome proliferator-activated receptor gamma coactivator 1 alpha (PGC-1α), a master regulator of mitochondrial biogenesis and function. This suppression leads to reduced mitochondrial content and compromised oxidative metabolism. In S6K1-knockout mice, enhanced PGC-1α expression correlates with increased mitochondrial biogenesis and improved metabolic health [34]. The role of S6K1 extends to models of mitochondrial disease. NADH dehydrogenase [ubiquinone] iron–sulfur protein 4 (Ndufs4) is a critical subunit of mitochondrial complex I, essential for proper electron transport chain function. Indeed, Ndufs4-knockout (NKO) mice have severe complex I deficiency [35]. Notably, in NKO mice, whole-body deletion of S6K1 resulted in a modest extension of lifespan and a delay in the onset of neurological symptoms. Interestingly, liver-specific deletion of S6K1 produced similar benefits, whereas deletion in brain or adipose tissue did not yield significant improvements. This suggests that S6K1 activity in the liver can influence the progression of mitochondrial diseases, potentially through systemic metabolic effects. The findings indicate that mTOR/S6K1 signaling can modulate mitochondrial disease outcomes via cell non-autonomous mechanisms [35]. Beyond mitochondrial dynamics, S6K1 has been implicated in broader metabolic regulation during aging. S6K1-knockout mice not only have an extended median and maximum lifespan, but they are protected against age- and diet-induced obesity and exhibit enhanced insulin sensitivity [13,36]. Specifically, Thomas and colleagues reported that S6K1-KO mice are smaller than wild-type mice when kept on a regular diet, which seems associated with a lower body fat index. Notably, S6K1 KO mice were resistant to diet-induced obesity [36]. S6K1 KO mice also display increased mitochondrial content and oxidative capacity in skeletal muscle, contributing to improved metabolic profiles [36]. This underscores the role of S6K1 in energy balance and metabolic health, which are critical factors in the aging process.

Hyperactivation of S6K1 has been linked to oxidative stress and endothelial dysfunction, common features of vascular aging. In senescent endothelial cells, increased S6K1 activity correlates with elevated superoxide production and decreased bioactive nitric oxide (NO) levels, indicative of endothelial dysfunction. Silencing S6K1 in these cells reduces superoxide generation and enhances NO production. Conversely, overexpression of a constitutively active S6K1 induces premature senescence and endothelial dysfunction through endothelial nitric oxide synthase (eNOS) uncoupling. These findings suggest a causal role for hyperactive S6K1 in promoting oxidative stress and vascular aging [30]. Consistent with these observations, overactivation of S6K1 has been associated with insulin resistance in skeletal muscle and in adipose tissue [37,38]. In summary, S6K1 plays a multifaceted role in regulating mitochondrial function and energy metabolism, with significant implications for cellular aging. Its influence on mitochondrial dynamics, metabolic pathways, and oxidative stress underscores its potential as a therapeutic target for mitigating age-related mitochondrial dysfunction and associated diseases.

### 2.3. S6K1 and Autophagy Decline in Aging

Autophagy is a fundamental cellular process responsible for the degradation and recycling of damaged organelles and misfolded proteins, thereby maintaining cellular homeostasis and energy balance [39]. The process initiates with the formation of an isolation membrane, known as the phagophore, which engulfs cytoplasmic components to form a double-membraned vesicle called the autophagosome. This process is tightly regulated by the unc-51-like autophagy-activating kinase 1 (ULK1) complex, which includes ULK1, autophagy-related proteins (ATG) 13 and 101, and focal adhesion kinase family-interacting protein of 200 kDa (FIP2000). Upon activation, ULK1 phosphorylates downstream targets, including components of the class III phosphatidylinositol 3-kinase (PI3K) complex, such as Beclin-1 and vacuolar protein sorting 34 (VPS34), to facilitate phagophore expansion [40].

The elongation and closure of the autophagosome require two ubiquitin-like conjugation systems involving other ATG proteins. The ATG12-ATG5-ATG16L1 complex facilitates membrane elongation, while the lipidation of microtubule-associated protein light chain 3 (LC3-I) to LC3-II allows for autophagosome membrane expansion and cargo recruitment [40]. The adaptor protein p62 plays a key role in selectively delivering ubiquitinated proteins and dysfunctional organelles to the autophagosome by interacting with LC3-II [41]. Following the formation of the autophagosome, it fuses with a lysosome to form an autolysosome, where lysosomal hydrolases degrade the cargo, and the breakdown products are recycled back into the cytoplasm for reuse [40]. This tightly regulated process is crucial for cellular adaptation to stress conditions, such as nutrient deprivation, and plays a significant role in development, differentiation, and the prevention of various diseases. Dysregulation of these key molecular players contributes to aging-associated impairments in autophagic flux, ultimately leading to the accumulation of damaged proteins and organelles, increased oxidative stress, and cellular dysfunction. Indeed, autophagy dysfunction has been linked to aging and several neurodegenerative diseases, including AD [41].

S6K1 has been directly implicated in the regulation of autophagy. For instance, the absence of S6K1 in MEFs impairs autophagic flux under stress conditions. Specifically, S6K1-deficient cells exhibit disrupted microtubule acetylation, which is essential for the proper transport and fusion of autophagosomes with lysosomes, thereby hindering autophagosome maturation and degradation of autophagic cargo. Reintroduction of S6K1 in these cells restores tubulin acetylation and normal autophagic flux, highlighting the indispensable role of S6K1 in stress-induced autophagy [42].

In human prostate cancer PC-3 cells, overexpression of constitutively active S6K1 has been shown to decrease levels of LC3-II, a marker of autophagosomal membranes, and reduce the formation of autophagic vacuoles in response to sulforaphane, an autophagy inducer. Conversely, depletion of S6K1 leads to the accumulation of autophagosomes and a decrease in autophagolysosome numbers, indicating a disruption in autophagic flux. These findings suggest that while S6K1 activity can suppress autophagosome formation, it is also necessary for the maturation and completion of the autophagic process [43].

The dysregulation of S6K1 and subsequent impairment of autophagy have significant implications for aging. Hyperactive S6K1 signaling has been associated with oxidative stress and endothelial dysfunction, common features of vascular aging. In senescent endothelial cells, increased S6K1 activity correlates with elevated superoxide production and decreased bioactive nitric oxide levels, indicative of endothelial dysfunction. Silencing S6K1 in these cells reduces superoxide generation and enhances nitric oxide production, suggesting a causal role of hyperactive S6K1 in promoting oxidative stress and vascular aging [30].

Under nutrient-rich conditions, mTORC1 is active and phosphorylates S6K1, which in turn promotes protein synthesis and cell growth while inhibiting autophagy. Conversely, during nutrient deprivation, mTORC1 activity declines, resulting in reduced S6K1 phosphorylation and activation of autophagic processes, indicating that S6K1 acts as a key mediator linking nutrient sensing to autophagy regulation [30,44].

However, accumulating evidence indicates that the role of S6K1 in autophagy is not unidirectional and under certain conditions, S6K1 may actually promote autophagy. For example, in macrophages and cancer cells, S6K1 enhances lysosomal biogenesis and autophagic capacity by supporting transcription factor EB (TFEB) activation and modulating the translation of key autophagy regulators [45]. In hepatic and skeletal muscle cells, S6K1 has been shown to facilitate mitophagy and starvation-induced autophagy, suggesting that it may act as a context-specific regulator, particularly under transient stress or during metabolic remodeling [46,47,48]. Consistent with these observations, pharmacological modulation of S6K1 further supports its role in autophagy control. For example, resveratrol, a polyphenolic compound known to inhibit S6K1, has been shown to suppress autophagy under conditions of nutrient limitation or rapamycin treatment in various cell lines, an effect attributed to the inhibition of S6K1 signaling [44]. These findings highlight the context-dependent role of S6K1 in autophagy, where its activity may either promote or inhibit autophagic flux depending on cellular and environmental cues. Overall, growing evidence cell-type-specific roles of S6K1 may determine its net effect on autophagy in the brain. In microglia, for instance, S6K1 appears to support autophagy-mediated clearance of pathological substrates and enhances lysosomal acidification, which may contribute to protective immune responses in neurodegenerative conditions [49]. These divergent roles emphasize that S6K1-mediated autophagy is finely tuned by factors such as nutrient availability, AMPK/mTOR balance, and subcellular localization of signaling components.

In summary, S6K1 plays a multifaceted role in regulating autophagy, acting as both a suppressor and a facilitator depending on the context. Its proper function is essential for maintaining cellular homeostasis, and its dysregulation contributes to aging-related pathologies. Understanding the nuanced relationship between S6K1 activity and autophagy is crucial for developing therapeutic strategies aimed at mitigating age-associated diseases.

### 2.4. S6K1, Insulin Resistance, and Aging

Aging is characterized by progressive metabolic decline, which manifests as impaired glucose homeostasis, reduced insulin sensitivity, mitochondrial dysfunction, and increased lipid accumulation [50,51,52]. These alterations contribute to a variety of age-related disorders, including type 2 diabetes, cardiovascular disease, and neurodegenerative diseases such as AD. A key feature of metabolic aging is insulin resistance, a condition where cells fail to respond adequately to insulin signaling, leading to hyperglycemia and compensatory hyperinsulinemia [53]. This metabolic imbalance exacerbates systemic inflammation, oxidative stress, and cellular senescence, all of which accelerate aging-related pathology [54,55]. At the molecular level, insulin signaling is primarily mediated by the PI3K-Akt pathway, which activates downstream effectors such as the mTORC1/S6K1 signaling axis [29]. While transient activation of mTORC1/S6K1 supports cellular growth and metabolic regulation, chronic hyperactivation contributes to insulin resistance and metabolic aging [29]. A key mechanism involves S6K1-mediated phosphorylation of insulin receptor substrate-1 (IRS-1) on specific serine residues (e.g., Ser302, Ser636/639), promoting IRS-1 degradation and subsequent attenuation of insulin signaling [29]. Evidence from both genetic and pharmacological models supports the role of S6K1 as a negative regulator of insulin sensitivity. For example, S6K1-knockout mice show enhanced insulin signaling, increased Akt phosphorylation, and improved glucose homeostasis [36], while inhibition of S6K1 with rapamycin or genetic deletion prevents IRS-1 degradation and restores insulin responsiveness [56]. These findings underscore the central role of S6K1 in metabolic decline associated with aging and highlight its potential as a therapeutic target. Converging evidence indicates that persistent S6K1 activation results in sustained IRS-1 inhibition, contributing to insulin resistance. For example, S6K1-KO mice exhibit enhanced insulin sensitivity, reduced fasting glucose levels, and improved glucose tolerance. Mechanistically, this is attributed to preserved IRS-1 signaling, increased Akt phosphorylation, and enhanced glucose uptake in insulin-sensitive tissues [36]. Along these lines, in hepatocytes and adipocytes, S6K1 overactivation leads to excessive IRS-1 phosphorylation and degradation, impairing downstream insulin signaling. Conversely, inhibition of S6K1 with rapamycin or genetic deletion of S6K1 restores IRS-1 stability and enhances insulin responsiveness [56]. These and other findings establish S6K1 as a negative regulator of insulin signaling, with its chronic activation contributing to age-related metabolic dysfunction. Taken together, these studies clearly indicate that chronic S6K1 activation contributes to insulin resistance, mitochondrial decline, and lipid accumulation, exacerbating age-related metabolic disorders. Given its central role in metabolic regulation, S6K1 represents an attractive target for therapeutic interventions aimed at promoting healthy aging and preventing metabolic diseases.

### 2.5. S6K1, Aging, and Inflammation

Chronic low-grade inflammation, often referred to as “inflammaging,” is a hallmark of aging and a key contributor to age-related functional decline and disease, including neurodegenerative disorders [57,58]. Increasing evidence suggests that S6K1 is a critical mediator of inflammatory responses during aging, acting through multiple mechanisms that converge on cytokine signaling, immune cell activation, and cellular stress pathways [59]. S6K1 modulates inflammation in part through its regulation of nuclear factor kappa B (NF-κB), a central transcription factor in immune and inflammatory signaling. S6K1 activity has been shown to promote NF-κB nuclear translocation and transcriptional activity via IκB kinase (IKK) complex activation [29]. In macrophages, genetic deletion or pharmacological inhibition of S6K1 reduces lipopolysaccharide (LPS)-induced secretion of pro-inflammatory cytokines such as tumor necrosis factor (TNF)-α, interleukin (IL)-6, and IL-1β, implicating S6K1 in innate immune activation [60]. Moreover, in aged tissues, elevated S6K1 signaling correlates with increased expression of senescence-associated secretory phenotype (SASP) factors, which include many inflammatory cytokines and chemokines [59]. Mechanistically, S6K1 has been shown to directly phosphorylate components of the Toll-like receptor (TLR) signaling cascade, including myeloid differentiation primary response protein 88 (MyD88) and tumor necrosis factor receptor-associated factor (TRAF6), thereby enhancing downstream activation of mitogen-activated protein kinase (MAPK) and NF-κB pathways [61]. Additionally, S6K1 influences NLR family pyrin domain-containing 3 (NLRP3) inflammasome activation. In metabolic tissues, S6K1 activation potentiates NLRP3-mediated caspase-1 activation and IL-1β maturation, linking nutrient sensing to innate immune responses [61]. Importantly, S6K1’s contribution to inflammation appears to be conserved across species. In Drosophila, reduced S6K signaling suppresses age-related inflammatory gene expression [7], while in mice, S6K1 knockout leads to decreased circulating inflammatory cytokines and improved tissue homeostasis in aging [59]. These findings suggest that S6K1 may act as a nodal regulator linking nutrient and stress signals to inflammatory aging pathways. Taken together, these data support a role for S6K1 in promoting age-associated inflammation through transcriptional, translational, and post-translational control of immune signaling networks. By exacerbating chronic inflammation, S6K1 may indirectly contribute to tissue damage and functional decline with age, positioning it as a potential therapeutic target for modulating inflammaging and its consequences. Overall, pharmacological and genetic studies suggest that reducing S6K1 activity can extend lifespan and improve health span. For instance, deletion of S6K1 in mice enhances metabolic flexibility, increases mitochondrial function, and improves cognitive performance [36,62,63,64,65]. Rapamycin, an mTOR inhibitor, has also been demonstrated to promote longevity by suppressing S6K1 activity [13]. Understanding the molecular mechanisms underlying these effects will be crucial for developing targeted therapies against aging and age-related diseases, including AD. In this section, we detailed the molecular underpinnings of S6K1’s role in aging, highlighting its regulation of protein synthesis, mitochondrial function, autophagy, and metabolic signaling. In the next section, we will explore how these processes intersect with AD pathogenesis and whether targeting S6K1 could provide neuroprotective benefits.

## 3. S6K1 in the Nervous System

### 3.1. Expression and Function of S6K1 in Neurons and Glial Cells

S6K1 is widely expressed in the central nervous system, where it functions as a key effector of the mTORC1 signaling pathway in both neurons and glial cells. Its activation is primarily controlled by phosphorylation at Thr389 by mTORC1, with additional modulation by PDK1 at Thr229, ensuring full enzymatic activity [66]. In neurons, S6K1 localizes to dendritic spines and axon terminals, where it regulates local protein synthesis essential for synaptic plasticity. Through its phosphorylation of ribosomal protein S6 (Ser235/236, Ser240/244), eIF4B (Ser422), and eEF2K (Thr56), S6K1 promotes the translation of proteins critical for neuronal growth and function [13]. Additionally, S6K1 influences the expression of plasticity-related genes such as activity-regulated cytoskeleton-associated protein (Arc), brain-derived neurotrophic factor (BDNF), and Ca^2+^/calmodulin-dependent protein kinase II (CAMKII)-α, which are essential for synaptic remodeling and long-term potentiation (LTP) [65,67]. Knockout studies in mice have revealed that loss of S6K1 reduces dendritic spine density and the expression of synaptic proteins, including postsynaptic density (PSD)-95 and Synapsin-1, leading to impaired synaptic transmission and cognitive deficits [64].

In glial cells, S6K1 plays a crucial role in maintaining metabolic support for neurons. Astrocytes rely on S6K1 signaling to regulate glucose uptake via glucose transporter type 1 (GLUT1) and lactate production through lactate dehydrogenase (LDH)-A, which are essential for neuronal energy homeostasis. Additionally, S6K1 influences astrocytic glutamate clearance by modulating glial glutamate transporter type 1 (GLT-1) expression, preventing excitotoxicity in the brain [68]. In microglia, S6K1 is activated in response to inflammatory signals such as LPS and interferon-gamma (IFN-γ), leading to increased translation of IL-1β, TNF-α, and IL-6. It also phosphorylates IKKβ, a key component of the NF-κB pathway, enhancing the transcription of pro-inflammatory genes [69]. These findings highlight the role of S6K1 as a regulator of neuronal function and glial-mediated neuroinflammatory responses.

### 3.2. Impact on Synaptic Plasticity, Memory, and Learning

S6K1 is essential for the regulation of synaptic plasticity, memory, and learning. The activation of S6K1 during LTP facilitates the translation of synaptic proteins such as glutamate A1 (GluA1) and N-methyl-D-aspartate (NMDA) receptor subunits (NR2A), which are critical subunits of alpha-amino-3-hydroxy-5-methyl-4-isoxazolepropionic acid (AMPA) and NMDA receptors, respectively [70]. Experimental models of S6K1 deletion have shown that the loss of this kinase significantly reduces the levels of phosphorylated of S6 (Ser240/244), indicating impaired translational capacity at synapses. These deficits correlate with reduced expression of plasticity-related genes, including Arc, Homer1a, and early growth response 1 (Egr1), and an inability to induce LTP at hippocampal CA1 synapses [71]. Conversely, excessive S6K1 activity has been associated with enhanced long-term depression (LTD) due to increased translation of phosphatases such as protein phosphatase 2A (PP2A) and striatal-enriched protein tyrosine phosphatase (STEP), which dephosphorylate AMPA and NMDA receptors, weakening synaptic connections [72,73]. The role of S6K1 in cognitive function extends beyond synaptic plasticity, as pharmacological inhibition of S6K1 in the hippocampus, either through rapamycin or the selective inhibitor PF-4708671, impairs spatial learning in the Morris water maze and disrupts fear memory consolidation [74,75,76]. In the prefrontal cortex, S6K1 has been shown to regulate dopaminergic transmission, controlling the expression of dopamine receptors D1 and D2, both of which are involved in working memory and executive function. Dysregulation of this pathway has been implicated in cognitive impairment observed in neuropsychiatric disorders [77]. Collectively, these findings establish S6K1 as a crucial modulator of learning and memory by regulating synaptic translation, neurotransmitter signaling, and neuronal plasticity.

### 3.3. Evidence Linking S6K1 to Neuroinflammation and Cellular Stress Responses

Beyond its role in synaptic function, S6K1 contributes to neuroinflammatory responses and cellular stress adaptation. Microglial activation in response to injury or disease is tightly regulated by S6K1, as hyperactivation of this kinase enhances NF-κB signaling through phosphorylation of inhibitor of κB kinase β (IKKβ; Ser177/181), leading to increased production of inflammatory cytokines such as TNF-α, IL-6, and IL-1β [69]. In astrocytes, excessive S6K1 signaling promotes a shift toward a neurotoxic A1-like phenotype, characterized by reduced secretion of neuroprotective factors such as BDNF and glial cell line-derived neurotrophic factor (GDNF) and an increase in inflammatory mediators that contribute to neuronal damage [78]. Additionally, S6K1 has been implicated in the regulation of Toll-like receptor (TLR) signaling, particularly TLR4, which plays a role in chronic microglial activation observed in aging and neurodegenerative diseases [79,80].

Oxidative stress and mitochondrial dysfunction are also tightly linked to S6K1 activity. Overactive S6K1 signaling suppresses the transcriptional coactivator peroxisome proliferator-activated receptor gamma coactivator (PGC)-1α by phosphorylating it at Ser570, leading to decreased expression of antioxidant enzymes, including superoxide dismutase 2 (SOD2), nuclear factor erythroid 2-related factor 2 (Nrf2), and catalase [81]. This reduction in antioxidant defenses exacerbates reactive oxygen species (ROS) accumulation, contributing to neuronal damage. Additionally, hyperactivation of S6K1 has been shown to enhance NMDA receptor-mediated calcium influx, promoting excitotoxicity and mitochondrial depolarization, ultimately leading to apoptotic cell death [82]. Endoplasmic reticulum (ER) stress is another cellular process influenced by S6K1 activity. Chronic activation of S6K1 disrupts the PKR-like endoplasmic reticulum kinase (PERK)-eIF2α–activating transcription factor 4 (ATF4) signaling axis, reducing the expression of critical ER chaperones such as glucose-regulated protein 78 (GRP78) and X-box-binding protein 1 (XBP1), thereby impairing the brain’s ability to manage protein misfolding and increasing vulnerability to neurodegeneration [83]. The cumulative impact of these stress pathways highlights S6K1 as a central regulator of neuroinflammation and cellular homeostasis, suggesting that its dysregulation may exacerbate neurodegenerative processes during aging and disease.

## 4. S6K1 and Alzheimer’s Disease Pathogenesis

**Figure 2:**
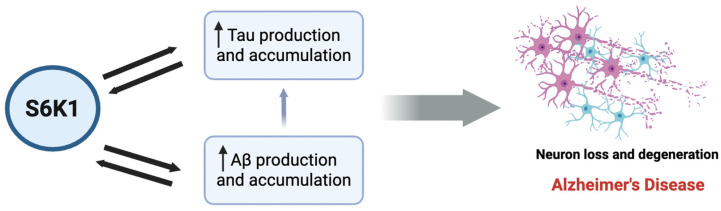
S6K1 interplay between Aβ and tau production and accumulation.

AD is characterized by progressive cognitive decline associated with the accumulation of amyloid beta (Aβ) plaques, tau hyperphosphorylation, synaptic dysfunction, and neurodegeneration [84]. The mTOR/S6K1 pathway has emerged as a critical regulator of AD pathogenesis, influencing Aβ metabolism, tau pathology, and synaptic plasticity (Figure 2) [85]. Hyperactivation of S6K1 has been implicated in multiple aspects of AD, contributing to neuronal stress, metabolic dysfunction, and cognitive impairment. We showed that in AD patients, the levels of phosphorylated S6K1 and its enzymatic activity were significantly increased compared to age-matched control cases [65]. Notably, we observed a positive correlation between S6K1 activity and total Aβ42 levels in AD brains and an inverse correlation between S6K1 activity and mini-mental state examination scores. These and other results suggest that Aβ accumulation may drive an increase in S6K1 activity (Figure 2) [65,86]. This section will explore the role of S6K1 in Aβ production and clearance, tau phosphorylation, and synaptic deficits in AD.

### 4.1. S6K1 and Aβ Pathology

#### 4.1.1. Regulation of APP Processing and Aβ Production

The amyloid precursor protein (APP) undergoes proteolytic processing through two major pathways, the non-amyloidogenic and amyloidogenic pathways, which determine whether Aβ peptides are produced. In the non-amyloidogenic pathway, APP is first cleaved by α-secretase (ADAM10 or ADAM17) within the Aβ domain, generating soluble APPα (sAPPα) and a membrane-bound C-terminal fragment (CTFα, C83), which is then further cleaved by γ-secretase into non-toxic peptides [87]. This pathway precludes Aβ formation and is considered neuroprotective.

Conversely, the amyloidogenic pathway is initiated by β-secretase (BACE1), which cleaves APP at the β-site, generating soluble APPβ (sAPPβ) and a C-terminal fragment known as C99 [87]. C99 is subsequently processed by the γ-secretase complex, composed of presenilin-1 (PSEN1), nicastrin, anterior pharynx defective-1 (Aph-1), and presenilin enhancer 2 (Pen-2), releasing Aβ peptides of varying lengths. Among these, Aβ42 is the most aggregation-prone and neurotoxic, forming oligomers and fibrils that contribute to plaque formation in AD [88]. The balance between these pathways is tightly regulated by intracellular signaling networks, including the mTOR/S6K1 pathway, which exerts significant control over APP processing and Aβ metabolism.

Hyperactivation of S6K1 promotes amyloidogenic APP processing by upregulating BACE1 expression and activity. Mechanistically, S6K1 phosphorylates forkhead box (Fox)-O3a at Ser253, leading to its nuclear exclusion and preventing its repression of BACE1 transcription [89]. This results in higher β-secretase activity and a corresponding rise in C99 and Aβ production. Furthermore, S6K1 enhances γ-secretase activity by modulating the stability and processing of PSEN1, further driving Aβ generation [90].

Additionally, the mTOR/S6K1 axis suppresses α-secretase activity, shifting APP processing toward the amyloidogenic pathway. This occurs through inhibition of ADAM10 transcription, mediated by reduced activation of the retinoic acid receptor β (RARβ), which is necessary for ADAM10 expression [91]. As a result, hyperactive S6K1 signaling favors Aβ production while reducing the formation of neuroprotective sAPPα fragments. Further supporting a role of S6K1 in APP processing, strong evidence indicates that S6K1 signaling influences γ-secretase activity by modulating the stability of PSEN1, the catalytic core of the γ-secretase complex. To this end, increased S6K1 activity correlates with enhanced γ-secretase-mediated cleavage of APP, further amplifying Aβ production [92]. Conversely, inhibition of S6K1 via rapamycin reduces Aβ levels in vitro and transgenic AD models, supporting its role in promoting amyloidogenic processing of APP [4,93].

#### 4.1.2. Autophagy-Mediated Clearance

Autophagy is another critical pathway for Aβ degradation, and S6K1 acts as a potent suppressor of autophagic flux. S6K1 inhibits autophagy by phosphorylating Unc-51-like kinase 1 (ULK1) at Ser757, preventing its activation by 5′-adenosine monophosphate (AMP)-activated protein kinase (AMPK) and blocking autophagosome formation [94]. In neurons, this inhibition leads to defective clearance of intracellular Aβ aggregates, which accumulate and contribute to neurotoxicity. Furthermore, S6K1 phosphorylation of TFEB at Ser211 retains it in the cytoplasm, preventing nuclear translocation and transcription of lysosomal genes required for autophagic degradation of Aβ [95]. Further, S6K1 may regulate autophagy indirectly through feedback loops involving AMPK, transcription factors like TFEB, and lysosomal biogenesis pathways [96]. Spatial transcriptomics studies discussed in Section 4.4 below show cell-type-specific dysregulation of autophagy-related and lysosomal genes in AD brains, reinforcing the importance of dissecting cell-specific S6K1 functions.

#### 4.1.3. Blood–Brain Barrier Transport

Aβ is also cleared from the brain via lipoprotein receptor-related protein 1 (LRP1), which facilitates its transport across the BBB into the circulation. S6K1 hyperactivation downregulates LRP1 expression by increasing levels of nuclear NF-κB, which represses LRP1 transcription [97]. This results in reduced efflux of Aβ from the brain, further contributing to its accumulation in AD.

### 4.2. S6K1 and Tau Pathology

Tau pathology, characterized by hyperphosphorylated tau protein forming neurofibrillary tangles (NFTs), is a major hallmark of AD. S6K1 plays a direct role in tau hyperphosphorylation by modulating multiple tau kinases. S6K1 activation enhances glycogen synthase kinase-3β (GSK-3β) activity by phosphorylating Ser9 on GSK-3β, preventing its inhibitory phosphorylation by protein kinase B (Akt) [98]. This increases tau phosphorylation at AD-associated epitopes, including Thr231, Ser396, and Ser404. In addition to GSK-3β, S6K1 signaling activates cyclin-dependent kinase 5 (CDK5), another key tau kinase. Hyperactivation of S6K1 promotes CDK5-mediated tau phosphorylation by increasing the expression of its activators, p35 and p25, through an NF-κB-dependent mechanism [99]. Furthermore, S6K1 directly phosphorylates tau at Ser262, a site known to reduce tau’s affinity for microtubules and promote its pathological aggregation [100]. In addition, elevated S6K1 activity exacerbates tau aggregation by impairing its clearance. Normally, tau degradation occurs through the ubiquitin–proteasome system (UPS) and autophagy–lysosomal pathways. However, S6K1 inhibits tau degradation by reducing autophagic flux, similar to its effects on Aβ clearance. Specifically, phosphorylation of TFEB (Ser211) by S6K1 prevents nuclear translocation of this master regulator of lysosomal biogenesis, reducing expression of key autophagy-related genes [101]. The resulting accumulation of hyperphosphorylated tau enhances its aggregation into NFTs, exacerbating neurodegeneration.

### 4.3. S6K1 and Synaptic/Cognitive Dysfunction

Tau synaptic dysfunction is an early and central feature of AD, contributing to cognitive impairment [102,103]. S6K1 activity modulates synaptic plasticity through its control of local protein synthesis. In AD models, hyperactivation of S6K1 has been linked to the excessive translation of Arc and Homer1a, leading to dysregulated AMPA receptor trafficking and synaptic depression [104]. This shift disrupts synaptic homeostasis, impairing LTP and promoting LTD, both of which are associated with memory deficits. Additionally, S6K1 hyperactivation suppresses BDNF-tropomyosin receptor kinase B (TrkB) signaling, which is essential for synaptic maintenance and plasticity. Increased S6K1 activity leads to phosphorylation-dependent degradation of IRS-1 (Ser636/639 phosphorylation), reducing Akt-mediated activation of cyclic AMP response element-binding protein (CREB), thereby impairing BDNF-induced gene expression [105]. Consequently, AD models with high S6K1 activity exhibit reduced dendritic spine density and impaired synaptic plasticity, both of which correlate with cognitive decline. Several studies have linked S6K1 hyperactivity to memory impairment in AD. In transgenic mouse models, pharmacological inhibition of S6K1 using PF-4708671 rescues memory deficits in contextual fear conditioning and novel object recognition tasks, suggesting that excessive S6K1 signaling contributes to cognitive dysfunction [104]. This rescue is accompanied by improved synaptic protein expression and enhanced LTP, further supporting the role of S6K1 in AD-related cognitive deficits. Moreover, S6K1 contributes to neuronal loss by promoting excitotoxicity and oxidative stress. Hyperactivation of S6K1 enhances NMDAR-mediated calcium influx, leading to mitochondrial dysfunction and activation of pro-apoptotic pathways, including Bax translocation to mitochondria and cytochrome c release, which ultimately result in caspase-dependent neuronal death [106]. This suggests that S6K1 not only disrupts synaptic function but also actively contributes to neurodegeneration in AD.

To further dissect the role of S6K1 in AD, we bred S6K1-knockout mice with 3xTg-AD mice, a widely used animal model of AD [65]. We provided compelling evidence that S6K1 plays a critical role in APP processing and Aβ pathology by modulating BACE1 expression and activity in vivo. Indeed, we reported that genetically reducing S6K1 led to enhancements in synaptic plasticity and spatial memory while also decreasing the accumulation of amyloid-β and tau. Mechanistically, these effects were associated with a reduction in the translation of tau BACE1 [65]. This study provides strong evidence that S6K1 is a critical regulator of Aβ pathology in AD. By modulating APP processing, BACE1 expression, and autophagic clearance, S6K1 inhibition emerges as a promising therapeutic strategy for reducing Aβ accumulation and rescuing cognitive decline in AD. The molecular mechanisms described in this section highlight S6K1 as a key modulator of AD progression, reinforcing the potential of targeting S6K1 as a therapeutic strategy. Future studies should further dissect the cell-type-specific contributions of S6K1 in AD, paving the way for novel pharmacological interventions aimed at modulating this pathway.

### 4.4. Cell-Type-Specific Role of S6K1 in Alzheimer’s Disease

In neurons, S6K1 regulates dendritic protein synthesis, synaptic plasticity, and memory consolidation [107]. Deletion of S6K1 in neurons enhances hippocampal-dependent learning and long-term potentiation [71]. S6K1 activity in neurons is tightly coupled to local translation in dendrites, particularly of mRNAs encoding synaptic proteins, and its aberrant activation in response to Aβ or tau pathology contributes to synaptic dysfunction in a mouse model of AD [65]. In astrocytes, S6K1 plays key roles in metabolic support of neurons, including lactate production and glutamate clearance. Alterations in astrocytic mTOR/S6K1 signaling can compromise lactate supply and impair glutamate uptake, potentially exacerbating excitotoxicity and neuronal stress [96]. Moreover, in astrocytes, S6K1 regulates the expression of inflammatory mediators, and its dysregulation may contribute to the chronic gliosis characteristic of AD.

In microglia, S6K1 modulates cytokine production, phagocytic activity, and metabolic reprogramming. S6K1-deficient microglia exhibit reduced expression of pro-inflammatory cytokines such as IL-1β and TNF-α, along with decreased oxidative stress under pathological conditions [7]. Given the central role of microglial activation in neuroinflammation and synaptic remodeling, cell-specific modulation of S6K1 in microglia may represent a promising therapeutic avenue in AD.

Three recent spatial transcriptomics and single-cell RNA-seq studies provide unprecedented resolution in mapping gene expression across brain cell types in aging and AD [108,109,110]. These studies reveal cell-type-specific and regionally distinct alterations in the expression of genes related to mTOR signaling, including S6K1 (encoded by RPS6KB1), and underscore the complexity of the cellular landscape that therapeutic interventions must navigate. Specifically, Mathys and colleagues used single-nucleus RNA-seq to profile over 80,000 nuclei from the prefrontal cortex of individuals across various stages of AD pathology. They identified widespread transcriptional dysregulation across multiple cell types, including excitatory neurons, microglia, and astrocytes. Pathway analysis revealed altered expression of genes involved in protein translation, immune activation, and stress responses, all of which are key processes downstream of mTOR/S6K1 signaling. Notably, some excitatory neuron subtypes showed significant downregulation of ribosomal and translation-associated genes, suggesting that early impairments in S6K1-mediated translational control may contribute to selective neuronal vulnerability [109]. Grubman and colleagues extended these findings to the entorhinal cortex, a region that is often affected early in AD. Their single-nucleus transcriptomic data identified cell-specific dysregulation in synaptic signaling, autophagy, and metabolism. Genes such as RPS6KB1, EIF4EBP1, and PIK3R1 showed altered expression in glutamatergic neurons and astrocytes. They also observed a reactive shift in astrocyte transcriptomes, characterized by increased expression of inflammatory and metabolic genes, processes strongly influenced by S6K1 [109]. Lau and colleagues combined spatial transcriptomics with single-nucleus RNA-seq to map the middle temporal gyrus and found that components of the mTOR pathway were differentially expressed in anatomically restricted glial subpopulations. These changes correlated with amyloid plaque burden and suggested region-specific alterations in autophagy, nutrient sensing, and immune activation, all potentially modulated by S6K1 [110].

Collectively, these findings reinforce that S6K1-related pathways are not uniformly dysregulated but instead show cell-type-specific and region-specific alterations in Alzheimer’s disease. This underscores the need for precision therapeutic strategies, where S6K1 modulation is tailored to the cellular and anatomical context to maximize efficacy and reduce off-target effects.

### 4.5. Sex-Dependent Differences in S6K1 Signaling in Alzheimer’s Disease

Sex differences in AD are well documented, with females showing higher incidence, faster cognitive decline, and distinct molecular pathology compared to males [111]. However, studies investigating sex-dependent regulation of the mTOR/S6K1 pathway remain limited. Emerging evidence from rodent models indicates that estrogen and other sex hormones modulate mTOR/S6K1 signaling in both the brain and peripheral tissues. For example, estradiol has been shown to activate the PI3K/Akt/mTOR pathway, leading to enhanced S6K1 phosphorylation in hippocampal neurons, which may contribute to neuroprotection and synaptic plasticity in females [112]. Conversely, age-related decline in estrogen levels may impair mTOR/S6K1 signaling in older females, possibly contributing to sex-specific vulnerability to AD pathology. One study by Brinton and colleagues reported sex-specific transcriptional responses in brain aging, with differences in insulin/mTOR signaling genes in female versus male mice [113]. Moreover, genetic deletion or pharmacological inhibition of S6K1 in mice has yielded divergent metabolic and cognitive outcomes depending on sex, although this has not been systematically examined in the context of AD [36,114].

## 5. S6K1 and Necroptosis: Implications for Alzheimer’s Disease

Necroptosis is a regulated form of programmed cell death that differs from apoptosis and is characterized by cellular swelling, membrane rupture, and the release of damage-associated molecular patterns (DAMPs) that trigger inflammation [115,116,117]. This pathway is primarily controlled by the receptor-interacting protein kinases (RIPK)-1 and RIPK3, along with their downstream effector, the mixed lineage kinase domain-like (MLKL) protein [118,119,120]. Under normal conditions, necroptosis is tightly regulated and serves as a defense mechanism against certain infections and stressors. However, in the context of neurodegeneration, dysregulated necroptotic signaling contributes to neuronal loss and chronic neuroinflammation.

In AD, increasing evidence suggests that necroptosis plays a role in neuronal death. We were the first to report that necroptosis is activated in postmortem AD brains. In addition, we showed that necroptosis activation positively correlated with Braak stage and negatively with both brain weight and cognitive scores [121]. This finding has been corroborated by others [122,123,124]. While the role of mTOR/S6K1 in apoptosis and autophagy is well documented, emerging studies indicate that S6K1 may also modulate necroptotic pathways. Specifically, S6K1 has been shown to interact with key regulators of necroptosis, particularly at the level of RIPK1. In some cellular contexts, mTOR/S6K1 signaling enhances RIPK1 stability and activity, thereby sensitizing cells to necroptotic death [125]. Additionally, S6K1 regulates protein synthesis and may influence the expression levels of necroptotic mediators such as RIPK3 and MLKL. A study demonstrated that inhibition of mTORC1/S6K1 signaling reduces RIPK3 expression and suppresses necroptotic cell death in inflammatory models [126]. These findings suggest that hyperactive S6K1 signaling, as seen in AD, could potentiate necroptotic cell death by increasing the availability or activation of necroptotic effectors.

Additionally, oxidative stress, a major driver of AD pathology, can amplify necroptotic signaling through S6K1-dependent mechanisms. S6K1 activation in response to oxidative stress has been shown to upregulate pro-inflammatory cytokines such as TNF-α [127], a well-known activator of RIPK1-dependent necroptosis. This pro-inflammatory environment further exacerbates neuronal loss and accelerates disease progression in AD. The intersection between S6K1 and necroptosis presents a novel therapeutic avenue for AD. Given that necroptosis contributes to neurodegeneration and that S6K1 activity is elevated in AD brains [65], inhibiting S6K1 may provide dual benefits by reducing both necroptotic cell death and neuroinflammation. Pharmacological inhibition of RIPK1, which prevents necroptotic signaling, has shown neuroprotective effects in AD models [128]. If S6K1 functions upstream of necroptosis by modulating RIPK1/RIPK3/MLKL signaling, targeting S6K1 may similarly reduce neuronal loss. Furthermore, given S6K1’s established role in Aβ and tau pathology [65], a therapeutic strategy that inhibits S6K1 could provide broader neuroprotection by simultaneously mitigating protein aggregation, neuroinflammation, and necroptotic cell death. Future studies should aim to clarify the precise molecular mechanisms linking S6K1 to necroptosis and explore whether S6K1 inhibitors can provide therapeutic benefits by preventing neuronal loss in AD.

## 6. S6K1 as a Therapeutic Target in Aging and Alzheimer’s Disease

### 6.1. Pharmacological and Genetic Strategies to Inhibit S6K1

Given the broad involvement of S6K1 in aging and AD pathogenesis, several pharmacological and genetic approaches have been explored to modulate its activity. One of the most well-established pharmacological strategies involves the use of mTORC1 inhibitors, such as rapamycin, which indirectly suppresses S6K1 activity. Rapamycin extends lifespan in multiple species, including yeast, worms, flies, and mice, partly through inhibition of S6K1-dependent protein synthesis and metabolic regulation [129]. Chronic rapamycin treatment has been shown to improve memory and synaptic plasticity in AD mouse models, reduce Aβ accumulation, and enhance autophagic clearance of toxic proteins [129]. However, because rapamycin inhibits the entire mTORC1 complex, its long-term use is associated with metabolic side effects, including dyslipidemia and immune suppression [130,131]. More selective S6K1 inhibitors have been investigated, such as PF-4708671, a compound that directly inhibits S6K1 catalytic activity without affecting mTORC1 or other kinases [132]. While PF-4708671 has shown promise in cancer models by reducing cell growth and proliferation, its effects on neuronal survival and cognitive function remain largely unexplored. Small-molecule inhibitors targeting S6K1’s ATP-binding pocket or its interaction with upstream regulators could offer novel therapeutic approaches with potentially fewer off-target effects. Genetic strategies have also provided critical insights into the potential of S6K1 suppression as a neuroprotective strategy. Mice with global S6K1 deletion exhibit extended lifespan, enhanced insulin sensitivity, and improved mitochondrial function [7]. As mentioned above, in the context of AD, genetic reduction of S6K1 improves cognitive function and synaptic plasticity while decreasing Aβ and tau pathology [65].

### 6.2. Effects of S6K1 Inhibition on Longevity and Neuroprotection

The beneficial effects of S6K1 inhibition on aging and neurodegeneration stem from its ability to regulate protein synthesis, mitochondrial metabolism, autophagy, and stress resistance. Studies in Drosophila have demonstrated that S6K1 inhibition increases lifespan and enhances neuronal resistance to oxidative stress by modulating the TOR-S6K1 signaling axis [133]. In mammals, inhibition of S6K1 promotes mitochondrial biogenesis and function, enhancing cellular energy production while reducing oxidative damage [36]. S6K1 suppression also exerts neuroprotective effects by enhancing autophagy, a process crucial for the clearance of toxic protein aggregates. In AD models, rapamycin treatment increases the turnover of dysfunctional mitochondria and Aβ oligomers via activation of autophagic pathways [134]. Similarly, genetic reduction of S6K1 enhances the degradation of hyperphosphorylated tau through increased lysosomal activity, protecting against neurofibrillary tangle formation [65]. These findings suggest that targeting S6K1 could mitigate multiple pathological features of AD by improving cellular quality control mechanisms.

### 6.3. Differentiating S6K1 and mTORC1 Effects on the Brain

As discussed above, mTORC1 is a central regulator of anabolic metabolism, acting upstream of several effectors, including S6K1, 4E-BP1, and ULK1 [96]. Pharmacological agents, such as rapamycin, inhibit mTORC1 activity and consequently reduce phosphorylation of downstream targets like S6K1. However, the effects of rapamycin are not limited to S6K1 inhibition: for instance, it reduces cap-dependent translation via 4E-BP1 and modulates autophagy through ULK1. Thus, some cognitive or neuroprotective effects attributed to mTOR inhibition may result from multi-effector actions, not exclusively from S6K1 modulation [45].

To separate these effects, genetic and pharmacological studies have employed S6K1-specific inhibition. For example, the small molecule PF-4708671 selectively inhibits S6K1 without affecting mTORC1 or 4E-BP1 [135]. As detailed above, when applied in preclinical models, PF-4708671 modulates protein synthesis and inflammatory responses more specifically than rapamycin [135]. Similarly, in S6K1-knockout mice, the beneficial effects on learning and memory are accompanied by unique transcriptional and synaptic signatures that are distinct from those observed in mTORC1-deficient models [65,67]. Furthermore, chronic mTORC1 inhibition via rapamycin is known to impair glucose metabolism, wound healing, and immune function [136]. In contrast, S6K1-deficient animals exhibit less pronounced metabolic side effects, suggesting a wider therapeutic window for S6K1-specific approaches [36].

### 6.4. Potential Benefits and Risks of Targeting S6K1 in AD Therapy

Targeting S6K1 presents a promising therapeutic avenue in AD, given its integral role in modulating cellular growth, protein synthesis, and metabolic signaling pathways implicated in neurodegeneration. In addition, selective inhibition of S6K1 holds potential to mitigate tau hyperphosphorylation, amyloid-β accumulation, and neuroinflammation, thereby preserving neuronal integrity and cognitive function. However, the therapeutic modulation of S6K1 is accompanied by complex challenges arising from its ubiquitous expression and pleiotropic functions, necessitating a nuanced understanding of its systemic and CNS-specific effects.

Chronic suppression of S6K1 activity has been shown to disrupt insulin signaling cascades, culminating in impaired glucose uptake and pancreatic β-cell dysfunction, which predispose to insulin resistance and type 2 diabetes mellitus. Given that metabolic syndrome and glucose dysregulation are established risk factors and exacerbators of AD pathology, it is imperative to rigorously assess the long-term metabolic consequences of S6K1-targeted therapies. Such adverse effects could paradoxically exacerbate neurodegenerative processes through vascular compromise and heightened oxidative stress.

As discussed above, within the CNS, S6K1 is involved in the regulation of synaptic protein synthesis, which is critical for synaptic plasticity, LTP, and memory consolidation. Experimental evidence indicates that S6K1 inhibition can enhance synaptic resilience under pathological conditions by modulating aberrant mTORC1 hyperactivity, thus improving neuronal survival and cognitive outcomes. However, S6K1 also facilitates normal synaptic transmission and adaptive plasticity, and indiscriminate or prolonged inhibition may impair these physiological processes, potentially leading to deficits in learning and memory in otherwise healthy neurons. Furthermore, the therapeutic window for S6K1 inhibition may differ substantially between young, aged, and diseased brains. In aging and AD, where mTOR/S6K1 signaling is aberrantly elevated, partial inhibition may restore balance, while in healthy systems, it may lead to underactivation of essential processes such as protein translation and autophagy.

Therefore, the development of therapeutic agents that selectively inhibit S6K1 within the brain, with fine-tuned activity profiles, is paramount. Strategies such as designing brain-permeable small molecules with allosteric modulatory effects, or employing targeted delivery systems (e.g., nanoparticle carriers or viral vectors), could maximize efficacy while minimizing peripheral metabolic disturbances. Furthermore, temporal modulation, allowing intermittent rather than continuous inhibition, may preserve essential S6K1 functions and reduce toxicity. To this end, a similar and successful approach has been used with mTOR inhibition [131]. Finally, biomarker-driven patient stratification and monitoring will also be critical to identify individuals who may benefit most from S6K1-targeted interventions, thereby enhancing clinical outcomes.

## 7. Open Questions and Future Directions

### 7.1. Remaining Gaps in Understanding S6K1’s Role in Alzheimer’s Disease

While substantial evidence supports a role for S6K1 in AD pathogenesis, several unanswered questions remain. First, it is unclear whether S6K1 activation is a primary driver of AD progression or if it merely amplifies disease-related pathways. Studies using inducible models of S6K1 overexpression in neurons and glial cells could help clarify its causal role in neurodegeneration. Second, the precise molecular mechanisms linking S6K1 to Aβ clearance and tau aggregation remain to be fully elucidated. While inhibition of S6K1 reduces BACE1 translation and tau synthesis, other downstream targets likely contribute to its neuroprotective effects. Proteomic and transcriptomic studies are needed to identify novel S6K1-regulated pathways in AD.

### 7.2. Need for In Vivo Validation and Clinical Translation

Although genetic and pharmacological studies in mouse models support S6K1 as a therapeutic target, in vivo validation in higher organisms is necessary. Non-human primate studies could provide insights into the effects of S6K1 modulation on cognitive function, metabolism, and neuronal integrity. Furthermore, clinical studies assessing S6K1 activity in human AD brains could establish its potential as a biomarker for disease progression. To translate these findings into clinical applications, researchers must develop safe and effective S6K1-targeting drugs. Current inhibitors, such as PF-4708671, have not been tested in human neurodegenerative diseases, and their pharmacokinetic properties must be optimized for brain penetration.

## 8. Conclusions

In this review, we highlighted the critical role of S6K1 in aging and AD, detailing its influence on protein synthesis, mitochondrial function, synaptic plasticity, and neuroinflammation. Emerging evidence suggests that pathological S6K1 activation exacerbates neurodegenerative processes, making it an attractive therapeutic target. Targeting S6K1 through pharmacological or genetic approaches has shown promise in preclinical models, improving cognitive function and reducing AD pathology. However, challenges remain, particularly regarding the long-term safety and specificity of S6K1 inhibition. Future studies should focus on identifying brain-selective inhibitors, elucidating mechanisms of S6K1 regulation in AD, and exploring potential combinatorial therapies. Ultimately, S6K1 represents a promising target for interventions aimed at mitigating the effects of aging and neurodegeneration. Continued research will be essential to determine whether modulating S6K1 activity can be translated into effective clinical therapies for AD and other age-related diseases.

## Data Availability

Not applicable.
